# Multilocus analysis of introgression between two sand fly vectors of leishmaniasis

**DOI:** 10.1186/1471-2148-8-141

**Published:** 2008-05-12

**Authors:** Camila J Mazzoni, Alejandra S Araki, Gabriel EM Ferreira, Renata VDM Azevedo, Guido Barbujani, Alexandre A Peixoto

**Affiliations:** 1 Laboratório de Biologia Molecular de Insetos, IOC, Fundação Oswaldo Cruz, Rio de Janeiro, Brazil; 2 Dipartimento di Biologia, Università di Ferrara, Ferrara, Italy; 3 Environmental Research Institute, University College Cork, Cork, Ireland; 4 present address: Department of Genetics, University of Leicester, Leicester, UK

## Abstract

**Background:**

The phlebotomine sand flies (Diptera:Psychodidae) *Lutzomyia (Nyssomyia) intermedia *Lutz & Neiva 1912 and *Lutzomyia (Nyssomyia) whitmani *Antunes & Coutinho 1932 are two very closely related species and important vectors of American cutaneous leishmaniasis. Two single-locus studies have revealed evidence for introgression between the two species in both mitochondrial and nuclear genomes. These findings have prompted the development of a multilocus approach to investigate in more detail the genetic exchanges between the two species.

**Results:**

We analyzed ten nuclear loci using the "isolation with migration" model implemented in the IM program, finding evidence for introgression from *L. intermedia *towards *L. whitmani *in three loci. These results confirm that introgression is occurring between the two species and suggest variation in the effects of gene flow among the different regions of the genome.

**Conclusion:**

The demonstration that these two vectors are not fully reproductively isolated might have important epidemiological consequences as these species could be exchanging genes controlling aspects of their vectorial capacity.

## Background

Gene flow between closely related species has been reported in an increasing number of studies as a rule and not as an exception and it is currently well accepted that sibling species can retain a low level of gene flow between them [1]. In this case, divergence between closely related species is determined by competition between gene flow and genetic drift, where the first tends to decrease divergence, and the latter to increase it [2]. A number of studies have provided evidence that introgression can occur more easily in certain regions of the genome. This is determined mainly by natural selection, which is expected to restrain gene flow at regions associated with species-specific adaptations [3].

In insect disease vectors, gene flow between species may have important epidemiological consequences, as it might allow changes in the disease patterns. Fonseca et al. [4] have shown that hybrids between two different forms in the *Culex pipiens *complex, probably introduced in the United States at two different moments, may act as a bridge vector between birds and humans of the West Nile virus, contributing to the current epidemics. In another example, Besansky et al. [5] have proposed that the acquisition of chromosome inversions by *Anopheles gambiae *from the more arid-adapted *Anopheles arabiensis *may have contributed to the spread and ecological dominance of this malaria vector.

Evidence of introgression has also been reported in sand flies [6,7], including *Lutzomyia intermedia *and *Lutzomyia whitmani *two important vectors of cutaneous leishmaniasis in Brazil [8]. Recently, we obtained further evidence for gene flow between these two closely related species using the *period *(*per*) gene [9], a locus controlling circadian and lovesong rhythms in *Drosophila*, that might have a role in the reproductive isolation between sibling species [reviewed in [10]]. These first pieces of evidence for introgression lead us to inquire about the level and extent of gene flow between *L. intermedia *and *L. whitmani*.

In this study, we performed a multilocus analysis using ten different nuclear loci in a sample of *L. intermedia *and *L. whitmani *from the locality of Afonso Claudio (Southeast Brazil) in order to estimate the level of gene flow between these two vector species in each individual locus and across the genome. The possibility that introgression between these two leishmaniasis vectors is extensive and recurrent, could have important epidemiological consequences. For example, genes controlling aspects of vectorial capacity such as adaptation to man made habitats (domestic and peridomestic areas), competence to transmit different *Leishmania *strains and host preference could be passing from one species to another [8]. Analysis of multiple markers might determine if introgression between the two species is affecting many loci and whether gene flow in genes known to control aspects of the reproductive isolation in other species, such as *period*, is relatively reduced compared to other loci.

## Results

### Molecular markers

The choice of molecular markers includes genes with different functions and genome locations as described in the Methods. The ten loci used in this study are the homologues of the following *Drosophila *genes: *Ca1D *(or *Ca-α1D*; Ca^2+^-channel protein α_1 _subunit D), *cac *(*cacophony*), *Rp49 *(or *RpL32*; *Ribosomal protein L32*), *RpL17A *(or *RpL23*; *Ribosomal protein L23*), *RpL36 *(*Ribosomal protein L36*), *RpS19a *(*Ribosomal protein S19a), TfIIA-L *(*Transcription factor IIA L*), *up *(*upheld*) and *ζCOP *(or *zetacop*) [11]. In addition, we also obtained new sequences of *per *from the same samples. Sequences have been submitted to Genbank (accession numbers EU082834–EU083311).

### Polymorphism, Recombination and Divergence Analyses

Initially all sequences were checked for recombination, a necessary step for the IM analysis (see below). The four-gamete test [12] was carried out for each locus in order to identify fragments with no evidence for internal recombination events. The larger non-recombining (NR) block including at least part of an intron was finally chosen for each locus to be used in subsequent population analyses (see Methods).

Table 1 shows the minimum number of recombination events for each gene and summarizes polymorphism information for both the NR block and the whole fragment at each locus. It is noticeable that some of the loci have a large difference in length and in the number of segregating sites between the whole fragment and the NR block, but this is often due to the high number of recombination events identified in some loci. However, in general, this difference does not affect the level of per nucleotide polymorphism, except for an increase of π and θ in the *per *and *cac *NR blocks, which is probably due to differences in evolutionary rates between different parts of these gene fragments.

**Table 1:** Polymorphism analyses for each locus/non-recombining block

locus	species	n^a^	Length(bp)	RM^b^	S^c^	π^d^	θ^e^	Tajima's D^f^
*Ca1D*	*L.intermedia*	27(25)	176(176)	1	6(5)	0.0141(0.0129)	0.0088(0.0075)	1.7251(2.0390)
	*L.whitmani*	28(24)			13(11)	0.0205(0.0185)	0.0190(0.0167)	0.2687(0.3541)
*cac*	*L.intermedia*	12(11)	172(51)	2	4(3)	0.0100(0.0235)	0.0077(0.0201)	1.0291(0.5873)
	*L.whitmani*	30(28)			9(8)	0.0104(0.0306)	0.0132(0.0403)	-0.6517(-0.7434)
*per*	*L.intermedia*	21(20)	481(86)	13	29(7)	0.0154(0.0204)	0.0168(0.0229)	-0.4254(-0.3674)
	*L.whitmani*	23(22)			36(12)	0.0236(0.0312)	0.0203(0.0383)	0.2943(-0.8805)
*Rp49*	*L.intermedia*	24(18)	237(237)	5	13(12)	0.0149(0.0135)	0.0147(0.0147)	0.0440(-0.3030)
	*L.whitmani*	17(16)			5(5)	0.0040(0.0035)	0.0062(0.0064)	-1.1412(-1.4912)
*RpL17A*	*L.intermedia*	20(16)	238(114)	5	13(5)	0.0188(0.0135)	0.0154(0.0132)	0.4893(0.0580)
	*L.whitmani*	31(31)			19(8)	0.0162(0.0101)	0.0200(0.0176)	-0.9231(-1.2859)
*RpL36*	*L.intermedia*	36(34)	412(113)	13	29(9)	0.0164(0.0185)	0.0170(0.0195)	-0.1108(-0.1489)
	*L.whitmani*	11(11)			29(6)	0.0279(0.0183)	0.0240(0.0181)	0.3898(0.0468)
*RpS19a*	*L.intermedia*	36(34)	194(117)	3	9(5)	0.0091(0.0106)	0.0112(0.0105)	-0.5708(0.0462)
	*L.whitmani*	48(46)			14(5)	0.0169(0.0119)	0.0163(0.0097)	-0.0895(0.0532)
*TfIIA-L*	*L.intermedia*	23(23)	355(254)	1	13(10)	0.0081(0.0080)	0.0099(0.0107)	-0.6493(-0.8337)
	*L.whitmani*	21(21)			11(11)	0.0115(0.0161)	0.0086(0.0120)	1.1776(1.1776)
*up*	*L.intermedia*	11(11)	428(354)	3	6(2)	0.0041(0.0010)	0.0048(0.0019)	-1.0943(-1.4296)
	*L.whitmani*	11(11)			22(14)	0.0172(0.0127)	0.0176(0.0135)	-0.4745(-0.7781)
*zetacop*	*L.intermedia*	29(28)	292(230)	3	10(8)	0.0056(0.0059)	0.0087(0.0089)	-1.1477(-1.0613)
	*L.whitmani*	19(18)			17(14)	0.0110(0.0116)	0.0167(0.0177)	-1.2924(-1.2976)

Note: values refer to sequences without gaps/ambiguous alignment; numbers in parentheses are related to the non-recombining block for each locus.

^a^Number of sequences for each species for each locus.

^b^The minimum number of recombination events.

^c^Number of segregating sites.

^d^The average number of nucleotide differences per site between two sequences.

^e^Theta (θ) = 4 N μ from S (number of segregating sites).

^f^None of the Tajima's D values was significant after Bonferroni's correction.

Divergence between *L. intermedia *and *L. whitmani*, estimated from *F*_*st *_values, is relatively high (Table 2), ranging from 0.17 to 0.64, except for *zetacop*, which shows limited divergence between the two species (*F*_*st *_= 0.0542 for the NR block, non significant). However, only three out of the ten loci present fixed differences (*Rp49*, *RpL36 *and *RpS19a*), whereas polymorphisms are shared in most genes, particularly in *per *(14 shared polymorphisms for the whole fragment). Some of the loci present differences in the degree of genetic divergence estimated from the analysis of the whole fragment and the NR block, respectively. The *RpL17A *NR block presents far less divergence between species compared to the whole fragment (*F*_*st *_= 0.1749 and 0.4135, respectively), while the opposite is observed in *RpL36 *(*F*_*st *_= 0.6026 and 0.3609 for the NR block and the whole fragment, respectively).

**Table 2:** Divergence between *L. intermedia *and *L. whitmani *at each locus, whole fragment and non-recombining block

locus	*F*_*st*_^a^	Nm^b^	Dxy^c^	Da^d^	Fixed^e^	Shared^f^
*Ca1D*	0.4139*(0.4567*)	0.354(0.2974)	0.0295(0.0289)	0.0122(0.0132)	0(0)	2(1)
*cac*	0.2874*(0.2593*)	0.6198(0.7142)	0.0143(0.0365)	0.0041(0.0095)	0(0)	1(1)
*per*	0.3188*(0.3313*)	0.5341(0.5045)	0.0288(0.0386)	0.0092(0.0128)	0(0)	14(1)
*Rp49*	0.6144*(0.6407*)	0.1569(0.1402)	0.0244(0.0236)	0.015(0.0151)	1(1)	1(0)
*RpL17A*	0.4135*(0.1749*)	0.3546(1.1798)	0.0301(0.014)	0.0126(0.0022)	0(0)	7(1)
*RpL36*	0.3609*(0.6026*)	0.4427(0.1649)	0.036(0.0464)	0.0138(0.028)	2(2)	6(0)
*RpS19a*	0.4593*(0.5618*)	0.2943(0.195)	0.024(0.0257)	0.011(0.0145)	1(1)	3(1)
*TfIIA-L*	0.4401*(0.3646*)	0.3181(0.4357)	0.0174(0.0188)	0.0076(0.0067)	0(0)	0(0)
*up*	0.3562*(0.4168*)	0.4518(0.3499)	0.0167(0.012)	0.0061(0.0051)	0(0)	3(1)
*zetacop*	0.0552^+^(0.0542^ns^)	4.2752(4.3621)	0.0088(0.0092)	0.0005(0.0005)	0(0)	3(1)

^a^Pairwise fixation index. Significance evaluated with 10000 permutations; * significant at P < 0.001;

^+ ^significant at P < 0.01; ^ns ^non significant P > 0.01.

^b ^Estimated number of migrants per generation between populations calculated from *F*_*st*_.

^c^The average number of nucleotide substitutions per site between populations.

^d^The number of net nucleotide substitutions per site between populations.

^e ^Number of fixed differences between species.

^f^Number of shared polymorphic sites between species.

### IM Analysis

An analysis of population divergence under the "Isolation with Migration" model has been performed using the IM software [2]. The IM analysis requires sequence data from individual loci that show variation within or between two populations, under the assumption that recombination is negligible. For this reason, only the NR blocks containing at least part of an intron have been used for the analyses and some putatively recombinant sequences were excluded (see Methods). In order to avoid mistaking the effects of selection for those of drift or migration, we carried out preliminary tests of selective neutrality. These included Tajima's D [13], Fu and Li's D* and F* [14], Ramos-Onsins and Rozas' R_2 _[15], and Fu's F_S _[16]. The statistic tests have been calculated using the DnaSP software [17] (for Tajima's D estimates see Table 1; for the other tests see additional file 1). The simulation analyses of the HKA software [18] were used for performing the HKA multilocus test of neutral molecular evolution [19]. In only one case (NR block of *zetacop *in *L. whitmani *for the F_S _test) a significant deviation from neutrality has been detected after Bonferroni's correction (see additional file 1). Based on their chromosome positions in *Drosophila *[11], all ten loci studied are expected to be unlinked, although we do not know their location in the *Lutzomyia *genome.

Migration parameters have been estimated for each locus as well as for all loci together for each population in different IM runs. Our aim was to detect the occurrence of gene flow using the multilocus data, and determine whether the evidence for introgression is exclusive to some loci. All marginal densities suggest stationary distributions, with one exception (see below).

**Figure 1:**
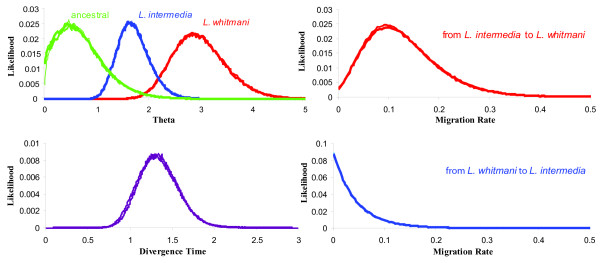
**Estimates of demographic parameters**. Marginal Posterior Probability Densities for each of the six demographic parameters estimated using IM: theta (*θ *= 4 N μ); migration rate (*m *= m/μ) and divergence time between species (*t *= t μ). Three IM simulations (a, b and c) with different seed numbers have been plotted for each parameter estimate (see also Table 3).

The marginal posterior probability densities for each of the six demographic parameters estimated using IM are shown in Figure 1. The results obtained suggest gene flow from *L. intermedia *to *L. whitmani *(Figure 1, top right graph). Table 3 summarizes the features from the marginal histograms for each of the parameters. The migration rate estimate with the highest smoothed value of likelihood is *m*_1 _= 0.095 (average of 3 independent runs) and the 95% confidence intervals exclude the value zero for gene flow (Table 3). On the other hand, no evidence of migration has been found in the other direction, from *L. whitmani *to *L. intermedia *(*m*_2 _= 0.002, that is *m*_2_~0 as 0.002 is the lowest value that can possibly be estimated by the program under the conditions used).

**Table 3:** Model parameter estimates for all loci

	*θ*_1_^a^			*θ*_2_^a^			*θ*_A_^a^			*t*^b^			*m*_1_^c^			*m*_2_^c^		
	a	b	c	a	b	c	a	b	c	a	b	c	a	b	c	a	b	c
Minbin^d^	1.236	1.263	1.453	0.714	0.790	0.790	0.014	0.014	0.014	0.578	0.593	0.588	0.002	0.002	0.002	0.002	0.002	0.002
Maxbin^d^	7.320	6.939	7.374	4.024	4.043	3.986	5.283	7.238	6.043	2.733	2.783	3.038	0.814	0.734	0.810	0.678	0.650	0.630
HiPt^e^	2.838	2.811	2.920	1.608	1.684	1.589	0.448	0.475	0.557	1.348	1.293	1.328	0.102	0.094	0.094	0.002	0.002	0.002
HiSmth^e^	**2.838**	**2.838**	**2.838**	**1.646**	**1.665**	**1.646**	**0.475**	**0.503**	**0.530**	**1.328**	**1.278**	**1.313**	**0.098**	**0.094**	**0.094**	**0.002**	**0.002**	**0.002**
Mean	2.947	2.947	2.947	1.703	1.703	1.684	0.638	0.665	0.638	1.338	1.323	1.323	0.118	0.114	0.114	0.030	0.030	0.030
95Lo^f^	2.078	2.078	2.105	1.189	1.189	1.189	0.041	0.068	0.041	0.918	0.913	0.908	0.018	0.022	0.018	0.002	0.002	0.002
95Hi^f^	4.115	4.142	4.142	2.407	2.407	2.407	1.833	1.833	1.860	1.848	1.828	1.843	0.302	0.298	0.294	0.174	0.170	0.170
HPD90Lo^g^	2.051	2.051	2.078	1.151	1.151	1.151	0.014	0.041	0.014	0.958	0.943	0.938	0.014	0.014	0.014	0.002	0.002	0.002
HPD90Hi^g^	3.870	3.897	3.925	2.274	2.274	2.274	1.399	1.426	1.426	1.743	1.718	1.723	0.242	0.238	0.238	0.110	0.106	0.110

Note: Values are presented for each of the three runs with different seed numbers (a, b and c).

^a ^The population size parameter for *L. whitmani *(*θ*_1_), *L. intermedia *(*θ*_2_) and ancestral population (*θ*_A_); *θ *= 4 Nm.

^b ^Time of population splitting parameter

^c ^Migration rate estimate (*m*_1 _– from *L. intermedia *to *L. whitmani*; *m*_2 _– from *L. whitmani *to *L. intermedia*).

^d ^The midpoint value of the lowest (Minbin) and highest (Maxbin) bin.

^e ^The value of the bin with the highest count (HiPt), after the counts have been smoothed by taking a running average of 9 points centered on each bin (HiSmth).

^f ^The estimated points to which 2.5% of the total area lies to the left (95Lo) and to the right (95Hi).

^g ^The lower (HPD90Lo) and upper (HPD90Hi) bound of the estimated 90% highest posterior density (HPD) interval.

**Figure 2:**
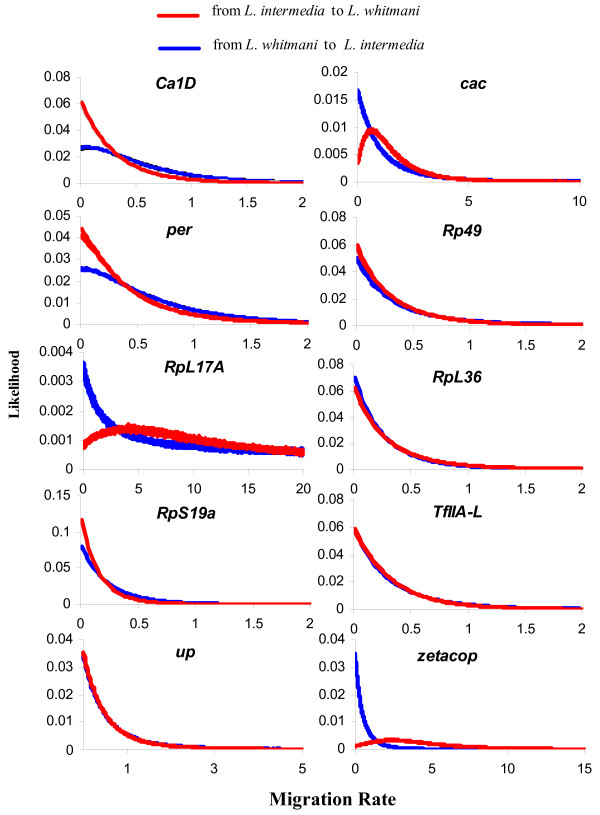
**Migration rate estimates in 10 different loci**. Marginal Posterior Probability Densities for each locus migration rate. Four IM simulations with different seed numbers have been plotted.

Simulations were also carried out to estimate the migration rates of each locus separately. Figure 2 shows the marginal posterior probability densities for each locus migration rate while Table 4 presents a summary from the marginal histograms. The results have revealed nonzero values in three different loci, *cac*, *RpL17A *and *zetacop *towards *L. whitmani *(Figure 2 and Table 4), showing no evidence of introgression between these two species at the other loci. Although the distribution for the *RpL17A *locus does not reach the zero value on its right tail, this gene shows the highest estimate for the migration parameter in the direction of *L. whitmani*.

**Table 4:** Migration parameter estimates for each locus

	***Ca1D***		***cac***		***per***		***Rp49***		***RpL17A***		***RpL36***		***RpS19a***		***TfIIA-L***		***up***		***zetacop***	
	*I*	*W*	*I*	*W*	*I*	*W*	*I*	*W*	*I*	*W*	*I*	*W*	*I*	*W*	*I*	*W*	*I*	*W*	*I*	*W*
Minbin^a^	0.01	0.01	0.01	0.01	0.01	0.01	0.01	0.01	0.01	0.01	0.01	0.01	0.01	0.01	0.01	0.01	0.01	0.01	0.01	0.01
Maxbin^a^	13.54	12.21	19.99	19.99	15.93	12.93	19.99	18.91	19.99	19.99	16.45	15.88	5.45	4.04	15.37	9.87	19.99	19.99	19.99	19.99
HiPt^b^	0.07	0.01	0.01	0.64	0.04	0.01	0.01	0.01	0.03	4.26	0.01	0.01	0.01	0.01	0.01	0.01	0.01	0.01	0.01	2.41
HiSmth^b^	**0.05**	**0.01**	**0.01**	**0.63**	**0.02**	**0.01**	**0.01**	**0.01**	**0.01**	**3.95**	**0.01**	**0.01**	**0.01**	**0.01**	**0.01**	**0.01**	**0.01**	**0.01**	**0.01**	**2.48**
Mean	0.41	0.23	0.91	1.25	0.46	0.32	0.31	0.25	6.18	8.00	0.21	0.23	0.17	0.11	0.25	0.24	0.44	0.41	0.83	3.63
95Lo^c^	0.01	0.01	0.03	0.11	0.01	0.01	0.01	0.01	0.15	0.56	0.01	0.01	0.01	0.01	0.01	0.01	0.01	0.01	0.03	0.36
95Hi^c^	1.98	1.38	9.33	7.36	2.35	1.97	4.99	1.90	19.16	19.17	1.44	1.70	1.01	0.69	1.64	1.42	5.45	5.13	9.07	14.35
HPD90Lo^d^	0.01	0.01	0.01	0.01	0.01	0.01	0.01	0.01	0.01	0.01	0.01	0.01	0.01	0.01	0.01	0.01	0.01	0.01	0.01	0.01
HPD90Hi^d^	1.24	0.80	4.15	3.76	1.43	1.10	1.67	0.94	18.6?*	16.9?*	0.76	0.88	0.61	0.43	0.90	0.82	1.94	1.78	4.07	9.13

Note. I – *L. intermedia*; W – *L. whitmani*. Each estimated value is an average of four IM runs with different seed numbers.

^a ^The midpoint value of the lowest (Minbin) and highest (Maxbin) bin.

^b ^The value of the bin with the highest count (HiPt), after the counts have been smoothed by taking a running average of 9 points centered on each bin (HiSmth).

^c ^The estimated points to which 2.5% of the total area lies to the left (95Lo) and to the right (95Hi).

^d ^The lower (HPD90Lo) and upper (HPD90Hi) bound of the estimated 90% highest posterior density (HPD) interval.

* HPD estimate not reliable

Maximum likelihood estimates for *θ *suggest that the *L. whitmani *effective population size is almost twice as large as in *L. intermedia *(Figure 1) while the ancestral population is estimated to be smaller than the current ones, indicating a possible expansion for both species.

Divergence time as well as the other parameters estimated with IM cannot be directly converted to numbers in years or generations, since the mutation rates in these two species or in other sand flies are unknown. However, using *D. melanogaster *synonymous and nonsynonymous substitution rates for nuclear genes, 1.56 × 10^-8 ^and 1.91 × 10^-9 ^per site per year [20], respectively, seems to be a reasonable first step for estimating the divergence time between *Lutzomyia *species. Thus, our educated guess for the divergence time between *L. intermedia *and *L. whitmani *would be of approximately 800 thousand years.

### Genealogy Analysis

Gene trees for both entire sequence and NR block were estimated using Neighbor-Joining (NJ) and Parsimony methods, available in MEGA 3.1 [21], with similar results (data not shown). Only in the *RpL36 *gene tree the sequences of the two species occurred in two separate clusters (see below) while for 3 genes only one species formed a cluster (*RpS19a*, *TfIIa-L *and *Rp49*) and 5 genes did not present the sequences from any of the species in a single cluster (*Ca1D*, *cac*, *per*, *up *and *zetacop*) (data not shown).

**Figure 3:**
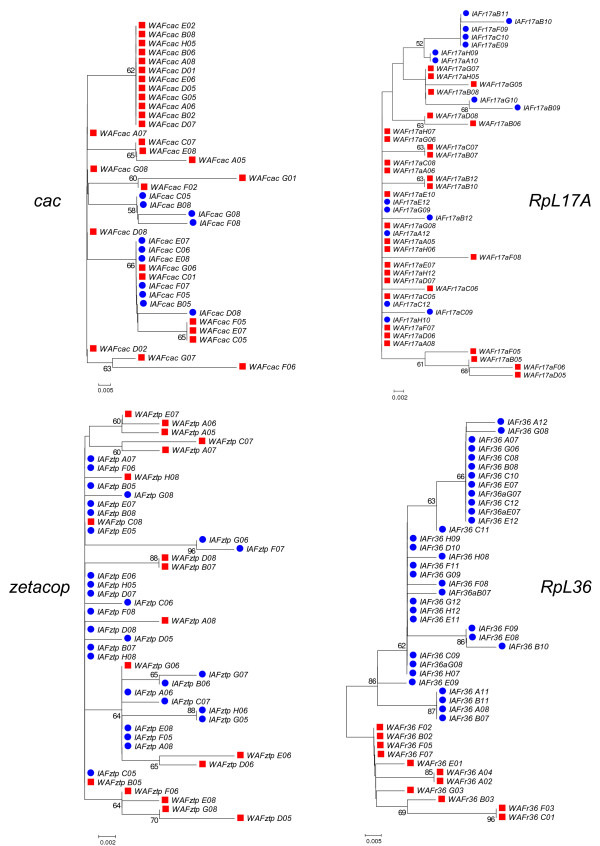
**Genealogies of NR blocks of four selected loci**. Genealogies from the NR blocks of the three loci with evidence of gene flow (*cac, RpL17A *and *zetacop) *and one locus (*RpL36*) presenting fixed differences between *L. intermedia *(blue circles) and *L. whitmani *(red squares). The trees were estimated using the neighbor-joining method, Kimura-2-parameters distance and 1000 bootstrap replicates. The trees were rooted using the middle-point between the two most distant sequences.

**Figure 4:**
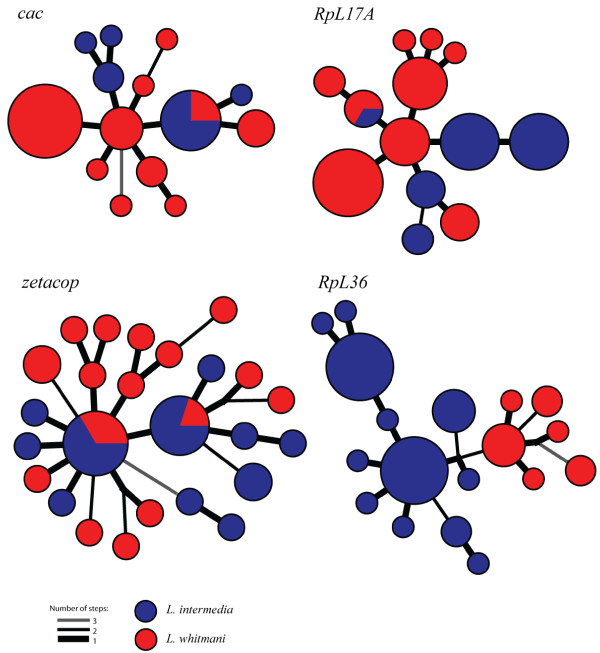
**Haplotype Networks of NR blocks of four selected loci**. Minimum spanning trees of the NR blocks of the three loci with evidence of gene flow (*cac, RpL17A *and *zetacop) *and one locus (*RpL36*) presenting fixed differences between *L. intermedia *(blue) and *L. whitmani *(red). The circles are proportional to the haplotype frequencies and the black and grey lines connecting the haplotypes represent the number of mutational steps.

Figure 3 presents the NJ trees from the NR blocks of the three loci that presented a nonzero migration estimate in the IM simulations: *cac*, *RpL17A *and *zetacop*. These trees are compared to the NJ tree of the *RpL36 *NR block (bottom right). While the former trees present non-structured topologies with a few identical haplotypes between the two species, the *RpL36 *NR block tree clearly groups both species in different clusters with a high bootstrap value (86%). Very similar results were obtained when haplotype networks were estimated using BioNumerics v. 5.0 (Applied Maths). Figure 4 shows Minimum Spanning Trees of *RpL36, cac*, *RpL17A *and *zetacop *NR blocks. Again only *RpL36 *presents a clear separation between the haplotypes of the two species.

## Discussion

Populations seldom evolve according to a simple and easy-to-identify mechanism. Under the model of Isolation with Migration, information on both divergence time and gene flow rates can be extracted from the data; thus, this model is more flexible and realistic than most alternative models of subdivided populations, which assume either absence of gene flow or an infinite divergence time [2,22]. Therefore, the IM model is particularly suitable for the study of recently-separated populations that may still be connected by some genetic exchanges, as we believe to be the case for the closely related species *L. intermedia *and *L. whitmani*, which despite presenting some identical haplotypes and no fixed differences in the *per *gene and in a mitochondrial marker [8,9] are nevertheless distinguishable by morphological differences [23]. Indeed, the simulations presented here between these two vectors showed good convergence and consistency among independent runs. In addition, the estimated population size difference between the species seems intuitively reasonable, since the distribution of *L. whitmani *is wider than *L. intermedia*. *L. whitmani *is distributed throughout most of Brazil occurring also in neighboring countries. *L. intermedia *occurs in the Northeastern and part of the Southeastern regions of Brazil [23-25].

Although genealogies can be difficult to interpret in case of recombination, phylogenetic analyses do not have to assume any historical demographical model [26], and therefore it is useful to observe the gene trees and compare them with our summary-statistics analyses. Figures 3 and 4 illustrate the difference between the topologies of loci presenting either evidence for gene flow or high differentiation between species.

Three loci present fixed differences between *L. intermedia *and *L. whitmani *and no evidence of gene flow and can therefore be suggested as molecular markers to differentiate the two species: *Rp49, RpS19a *and *RpL36*. The latter also presents insertions/deletions which have not been considered in Table 2. These three genes codify ribosomal protein subunits and it is intriguing that only such genes present fixed differences. As far as we know there is no reason one should expect these highly conserved ribosomal proteins to be less prone to introgression or to be under directional selection that would increase the likelihood of fixed differences between the two siblings. One interesting, but perhaps unlikely, possibility is that codon bias, as observed in *Drosophila *ribosomal protein genes [27], might somehow be responsible for the observed fixed differences.

The *cac *and *per *genes determine aspects of the lovesong patterns in *Drosophila *(reviewed in [28]), and therefore they are potentially good candidates for speciation loci in insects producing acoustic signals. So far, there is no behavioral study providing evidence that lovesongs play a role in the reproductive isolation between *L. intermedia *and *L. whitmani *as might be the case for the *Lutzomyia longipalpis s.l*. siblings [29,30]. Our data suggest the occurrence of introgression in *cac *and, although no evidence for gene flow in *per *was obtained in the present study, a previous analysis using this gene as a molecular marker also suggests introgression between *L. intermedia *and *L. whitmani *in at least one of the populations analyzed [9]. This evidence of gene flow in *cac *and *per *might indicate that these two loci do not have an important role, if any, in the reproductive isolation of *L. intermedia *and *L. whitmani*.

The *RpL17A *gene presented great differences between the genealogies and haploytpe networks obtained with the whole fragment and the NR blocks. While the latter show topologies with no grouping pattern for *L. intermedia *or *L. whitmani *and some identical haplotypes in both species (Figs. 3 and 4), the NJ tree and haplotype network from the whole fragment divide the species in two distinct groups (additional files 2 and 3). The same difference can be noticed for the *F*_*st *_values, 0.4135 and 0.1749 for the whole fragment and NR block, respectively (Table 2). The IM simulations using the NR blocks indicated migration in the direction of *L. whitmani *at this gene. The observation that non-recombining blocks present low-divergence between species in opposition to the whole gene, suggests the occurrence of migration followed by recombination, where new haplotypes are created by recombining different alleles inside a population instead of by the occurrence of new mutations. It also reinforces the idea that different regions of a gene might have different evolutionary histories [3].

The *zetacop *gene has also presented interesting results. The fragment analyzed here revealed a large level of gene flow between the two species, with low (whole fragment) or non significant values (NR block) of *F*_*st *_(Table 2) in addition to genealogies and networks that do not follow any grouping pattern (NR block in Figs. 3 and 4; whole fragment in additional files 2 and 3).

The epidemiological consequences of gene flow involving disease vectors are still not well known [31], however some studies do suggest important phenotypical changes in insect vectors due to introgression (see Background). The results obtained here are especially important because *L. intermedia *and *L. whitmani *are considered two main vectors of the cutaneous leishmaniasis, a disease for which the distribution has been expanding throughout Brazil, as silvatic areas have been constantly modified by urbanization [32-34]. The two species show some behavioral and ecological differences. *L. intermedia *is usually more common in the peridomestic area and more frequent in the summer months while *L. whitmani *is found mainly in the surrounding forest and is more abundant in the winter [33]. Interestingly it has been reported that only *L. whitmani *populations sympatric with *L. intermedia *are involved in cutaneous leishmaniasis transmission in the peridomestic environment [8]. The fact that we find evidence for introgression from *L. intermedia *towards *L. whitmani *is consistent with that although the loci we studied are probably not directly related to aspects of vectorial capacity. Further and more specific analyses focusing on the correlation between genetic, behavioral and habitat characteristics of these two sand fly species will be necessary to elucidate the possible consequences of gene flow for the disease epidemiology.

## Conclusion

In conclusion, we have found evidence of introgression from *L. intermedia *towards *L. whitmani *in three out of ten analyzed loci. In addition, a different study using the *per *gene to analyze the geographical variation among populations of the two species has also indicated introgression in this locus. These findings suggest the occurrence of gene flow in roughly one third of the genome of these two vectors of cutaneous leishmaniasis in Southeastern Brazil raising the question whether this might be related to the observed changes in the epidemiological patterns of this disease.

## Methods

### Choice of molecular markers

Four loci selected for this analysis (*Ca1D*, *cac*, *Rp49 *and *per*) had been previously isolated from *Lutzomyia *species in our lab [35-38]. To select additional molecular markers to use in our study, a screen of cDNA sequences of *L. longipalpis *available in our lab was carried out. Genes with high similarity at the protein level compared to *Anopheles gambiae *and/or *Drosophila melanogaster*, and which present at least one intron in the region of aligned fragments, have been preferentially selected under the assumption that they have a well known function and potentially present an intron in *Lutzomyia*. Out of the 280 cDNA sequences initially checked, 26 were selected for primer design for PCR amplification. After amplification tests using genomic DNA of *L. intermedia *and *L. whitmani*, we ended up with 6 loci (*RpL17A*, *RpL36*, *RpS19a, TfIIA-L*, *up, zetacop*). Primers sequences of all ten loci used in our multilocus analysis are presented in Table 5.

**Table 5:** List of primers and edition of sequences

locus	primers	Removed gap sites^a^	NR blocks^b^	Removed sequences from NR blocks^c^
*Ca1D*	5LWIca1D 5'-CAGGATATAATGATGGATTG-3' 3LWIca1D 5'-CACGAACAAGTTGATAAT-3'	159–160; 162–169; 183	1–176	IAFCa_A12; IAFCa_F09; WAFCa_B12; WAFCa_C09; WAFCa_H11; WAFCa_H12
*Cac*	5Llcac 5'-GTGGCCGAACATAATGTTAG-3' 3Llcac 5'-CCACGAACAAGTTCAACATC-3'	10; 19; 124–126; 129–132; 150; 170; 180; 182	122–172	IAFcac_H05; WAFcac_F01; WAFcac_H07
*Per*	5llper2 5'-AGCATCCTTTTGTAGCAAAC-3' 3llper2 5'-TCAGATGAACTCTTGCTGTC-3'	154–157; 167–169; 204	125–210	IAFPER16; WAFPER05
*Rp49*	5RP49semideg1 5'-TTCATYCGYCAYCAGWSBGA-3' 3llRP49exp2 5'-GGGCGATCTCAGCACAGTAT-3'	29–36; 46–49; 55–56; 59; 65–69; 72; 74; 87	1–237	IAFrp49_B08; IAFrp49_C08; IAFrp49_E05; IAFrp49_E08; IAFrp49_G08; IAFrp49_H06; WAFrp49_D11
*RpL17A*	5LLrpL17A 5'-TCAATTGCGCCGACAATAC-3' 3LLrpL17A 5'-GCTGATCCTTTCATTTCGCC-3'	90–91; 101–102; 109–110; 131; 135; 144; 147–148; 153–154	1–114	IAFr17a_D10; IAFr17a_E10; IAFr17a_F10; IAFr17a_G11
*RpL36*	5LWIrpL36 5'-GTTCCTCACGCTTCCTCTTG-3' 3LWIrpL36 5'-AAAGTGAAAGGACTCCGCCC-3'	26–29; 46; 52; 65; 77; 215–217; 226; 238–253; 267; 270; 274; 289–294; 362	126–238	IAFr36_D07; IAFr36_H11
*RpS19a*	5LWrpS19 5'-TGATCAACACAAGATTGTCCG-3' 3LWrpS19 5'-ACACCATTCCTCTTACGACC-3'	174	77–193	IAF19_G01; IAF19_H02; WAF19_B08; WAF19_F079
*TfIIA-L*	5LLTfIIA-L 5'-GATAATGATCCAGACGATGCC-3' 3LLTfIIA-L 5'-GAAAACATAGTCCTTCCCACC-3'	162; 173–179; 201–259; 277; 284–319; 341; 345–346; 369–370; 386–387	1–254	-
*Up*	5LLup 5'-GCAACAAGTCCAAAGAGCAG-3' 3LLup 5'-TCATAGGAGCGGGTGTCAAC-3'	189–191; 224; 351–355; 360–361; 368–369; 396–397	1–354	-
*zetacop*	5LLzetacop 5'-GGATGCAGATCCTTCATCCG-3' 3LLzetacop 5'-CGACCACTTCAGTTGTTCTC-3'	115; 131–134; 149–150; 168; 181; 195; 224; 234–235; 240–242	1–230	IAFztp_H07; WAFztp_C06

^a ^Sites with indels or ambiguous alignment removed before the analyses.

^b ^Fragment positions of the non-recombining blocks used in the analyses.

^c ^Recombining sequences removed before the analyses using the non-recombining blocks.

### Sand fly samples and DNA methods

Sand flies were collected in September 2004 from the locality of Afonso Claudio (Espírito Santo State, 20°04'S 41°07'W), Southeast of Brazil, where *L. intermedia *and *L. whitmani *occur in sympatry. This locality is far from the known range of *L. neivai*, a third sibling closely related to the other two species that occurs mainly in the Southern and Central Western regions of Brazil and in neighboring countries [8,24]. *L. intermedia *and *L. whitmani *were identified according to Young and Duncan [23]. DNA was extracted from 96 individuals from each species according to Jowett [39] with slight modifications. A mix including 1 μl of each individual DNA preparation was prepared for each species, and then used for PCR amplifications for all tested loci using Tth DNA polymerase (Biotools). PCR products were cloned in pMOS Blue Ended Vector (GE Healthcare) and sequenced at Fundação Oswaldo Cruz with an ABI 3730 DNA Sequencer, using "BigDye Terminators" (Applied Biosystems).

### Sequence analyses

Sequences were edited using Wisconsin Package Version 9.1, Genetics Computer Group (GCG) and aligned using ClustalX [40]. Sites within indels or ambiguous alignment were removed before the analyses (see Table 5). Polymerase error rate was estimated as 1.56 × 10^-3 ^per nt. Based on this error rate, putative PCR induced singletons were randomly selected and corrected. Analyses of population polymorphisms and differentiation between populations were carried out using DNAsp4.1 [17] and ProSeq [41] softwares. The former program was also used for the recombination analysis. Table 5 shows the position of the non-recombining (NR) blocks used in this study as well as the sequences removed before the analyses carried out using these fragments. Neutrality tests were carried out using the HKA software [18] and DNAsp4.1.

Markov Chain Monte Carlo (MCMC) simulations of the isolation with migration model were carried out using the IM program [2]. IM estimates the marginal posterior probability for 6 demographic parameters from multilocus data, using an implementation of the MCMC (Monte Carlo Markov Chain) algorithm: time of divergence between the species (*t*), effective population sizes for each species (*θ*_1 _and *θ*_2_) and the ancestral population (*θ*_A_), and migration rates in both directions (*m*_1 _and *m*_2_). The Infinite Sites model [42] has been chosen as the mutation model since these are closely related species and all genes are nuclear. Upper limits were set to prior values for each of the demographic parameters based on preliminary runs. All values in the range were considered to present equal probabilities. Runs have been repeated at least three times using different seed numbers and a large number of updates (~20 million) in order to guarantee convergence.

In addition to the simulations presented in the Results and Discussion section, tests using twice and half of the estimated number of singletons have been performed for IM analysis, in order to examine the effect of changes caused by differences in the number of corrected singletons. The results indicate small quantitative and no qualitative differences in the parameter estimates. The migration estimates did not change for *m2 *and show small differences for *m1*, around 0.018 in either direction.

## Authors' contributions

CJM, ASA, and RVDMA generated the data. CJM analyzed all the data and drafted the manuscript. ASA and GEMF collected sand fly samples. GB helped to write the manuscript and supervised CJM during her stay in Italy. AAP is the principal investigator, participated in its design and coordination, and helped to write the manuscript. All authors read and approved the final manuscript.

## Supplementary Material

Additional file 1 Supplemental Table – Tests of neutrality. The table presents the results of neutrality tests Fu and Li's D* and F*, Fu's F_S _and Ramos-Onsins and Rozas' R_2_. Click here for file

Additional file 2 Genealogies of *RpL17A *and *zetacop *whole fragments. The figure shows trees of *RpL17A *and *zetacop *sequences (whole fragments) of *L. intermedia *(blue circles) and *L. whitmani *(red squares). The trees were estimated using the neighbor-joining method, Kimura-2-parameters distance and 1000 bootstrap replicates, and rooted using the middle-point between the two most distant sequences. Click here for file

Additional file 3 Haplotype networks of *RpL17A *and *zetacop *whole fragments. The figure shows minimum spanning trees of *RpL17A *and *zetacop *sequences (whole fragments) of *L. intermedia *(blue) and *L. whitmani *(red). The circles are proportional to the haplotype frequencies and the black and grey lines connecting the haplotypes represent the number of mutational steps. Click here for file
