# Supplementary material for: Multilocus analysis of introgression between two sand fly vectors of leishmaniasis
Source: BMC Evol Biol. 2008 May 12;8:141. doi: 10.1186/1471-2148-8-141 (PMC2413237; doi:10.1186/1471-2148-8-141)
Supplement: Additional file 1 — Supplemental Table – Tests of neutrality. The table presents the results of neutrality tests Fu and Li's D* and F*, Fu's FS and Ramos-Onsins and Rozas' R2. [file 1471-2148-8-141-S1.doc]

# Additional file 1

**Supplemental Table – Tests of neutrality**

| locus | species | D*^a^ | F*^a^ | F_S_^b^ | R_2_^c^ |
| --- | --- | --- | --- | --- | --- |
| *Ca1D* | *L.intermedia* | 0.466 (1.162) | 0.973 (1.640) | 3.067 (6.398) | 0.202 (0.227) |
|  | *L.whitmani* | 0.200 (0.970) | 0.259 (0.916) | -1.731 (0.669) | 0.133 (0.145) |
| *cac* | *L.intermedia* | 0.368 (0.127) | 0.606 (0.271) | -1.748 (0.960) | 0.205 (0.195) |
|  | *L.whitmani* | 0.786 (-0.540) | 0.405 (-0.701) | -3.669 (-1.346) | 0.098 (0.109) |
| *per* | *L.intermedia* | -0.237 (0.048) | -0.342 (-0.080) | 0.640 (1.403) | 0.121 (0.127) |
|  | *L.whitmani* | 0.475 (-0.900) | 0.491 (-1.040) | -1.171 (-1.872) | 0.150 (0.109) |
| *Rp49* | *L.intermedia* | 0.259 (0.670) | 0.227 (0.455) | -4.479 (-0.931) | 0.129 (0.127) |
|  | *L.whitmani* | -1.097 (-1.808) | -1.273 (-1.974) | -2.698 (-1.924) | 0.097 (0.093) |
| *RpL17A* | *L.intermedia* | 0.778 (-0.297) | 0.806 (-0.231) | -2.144 (-1.417) | 0.166 (0.144) |
|  | *L.whitmani* | -0.172 (0.044) | -0.486 (-0.422) | -3.677 (-3.306) | 0.097 (0.071) |
| *RpL36* | *L.intermedia* | 0.780 (0.161) | 0.570 (0.076) | -17.264 (-0.899) | 0.113 (0.113) |
|  | *L.whitmani* | 0.637 (1.326) | 0.651 (1.134) | -1.869 (0.851) | 0.180 (0.173) |
| *RpS19a* | *L.intermedia* | 0.142 (1.132) | -0.095 (0.939) | -4.361 (-0.837) | 0.095 (0.124) |
|  | *L.whitmani* | -0.704 (0.335) | -0.588 (0.289) | -10.713 (-0.974) | 0.112 (0.138) |
| *TfIIA-L* | *L.intermedia* | -0.128 (-0.580) | -0.330 (-0.762) | -6.003 (-3.938) | 0.101 (0.093) |
|  | *L.whitmani* | 0.990 (0.990) | 1.212 (1.212) | 1.095 (1.095) | 0.182 (0.182) |
| *up* | *L.intermedia* | -0.712 (-1.658) | -0.912 (-1.797) | -5.035 (-1.246) | 0.117 (0.193) |
|  | *L.whitmani* | 0.351 (0.002) | 0.159 (-0.222) | -0.479 (-0.603) | 0.145 (0.134) |
| *zetacop* | *L.intermedia* | 0.340 (0.704) | -0.135 (0.203) | -2.077 (-0.859) | 0.079 (0.083) |
|  | *L.whitmani* | -0.561 (-0.587) | -0.896 (-0.913) | -10.632 (-8.527^#^) | 0.080 (0.083) |

^#^ significant after Bonferroni’s correction.

^a^ Fu and Li´s D* and F* [14].

^b^ Fu’s F_S_ [16].

^c^ Ramos-Onsins and Rozas’ R_2_ [15].
