# Supplementary figures and images for: Multilocus analysis of introgression between two sand fly vectors of leishmaniasis
Source: BMC Evol Biol. 2008 May 12;8:141. doi: 10.1186/1471-2148-8-141 (PMC2413237; doi:10.1186/1471-2148-8-141)

*RpL17A*

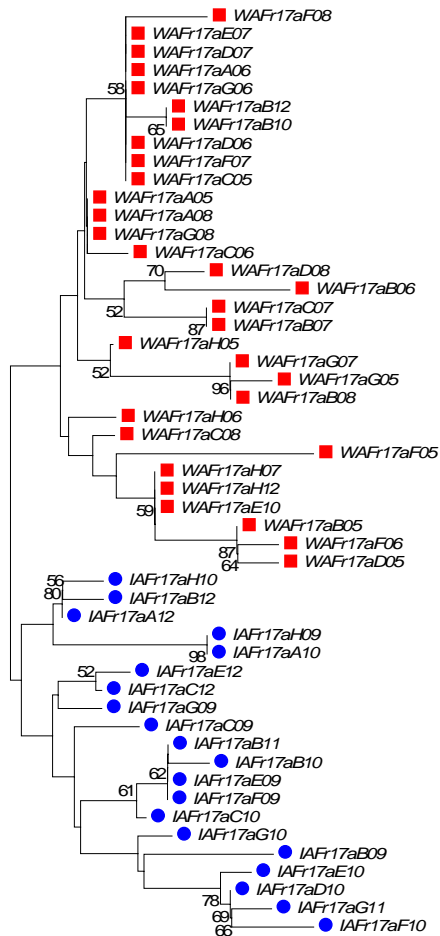

0.005

*zetacop*

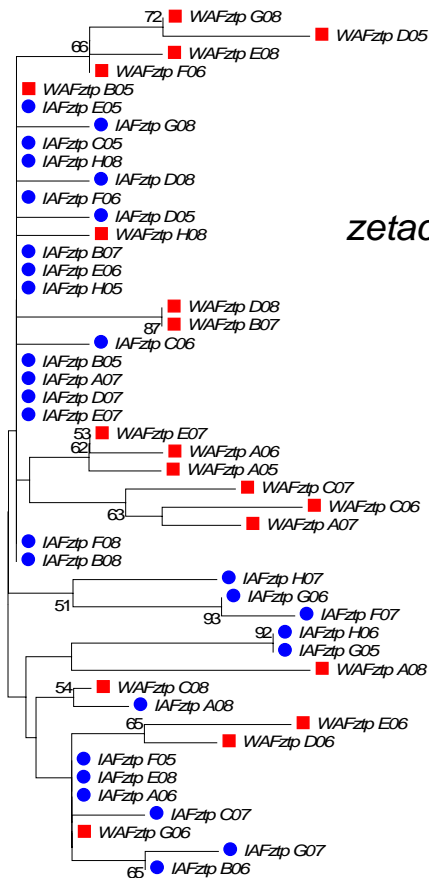

0.001

Supplement: Additional file 2 — Genealogies of RpL17A and zetacop whole fragments. The figure shows trees of RpL17A and zetacop sequences (whole fragments) of L. intermedia (blue circles) and L. whitmani (red squares). The trees were estimated using the neighbor-joining method, Kimura-2-parameters distance and 1000 bootstrap replicates, and rooted using the middle-point between the two most distant sequences. [file 1471-2148-8-141-S2.pdf]

*RpL17A*

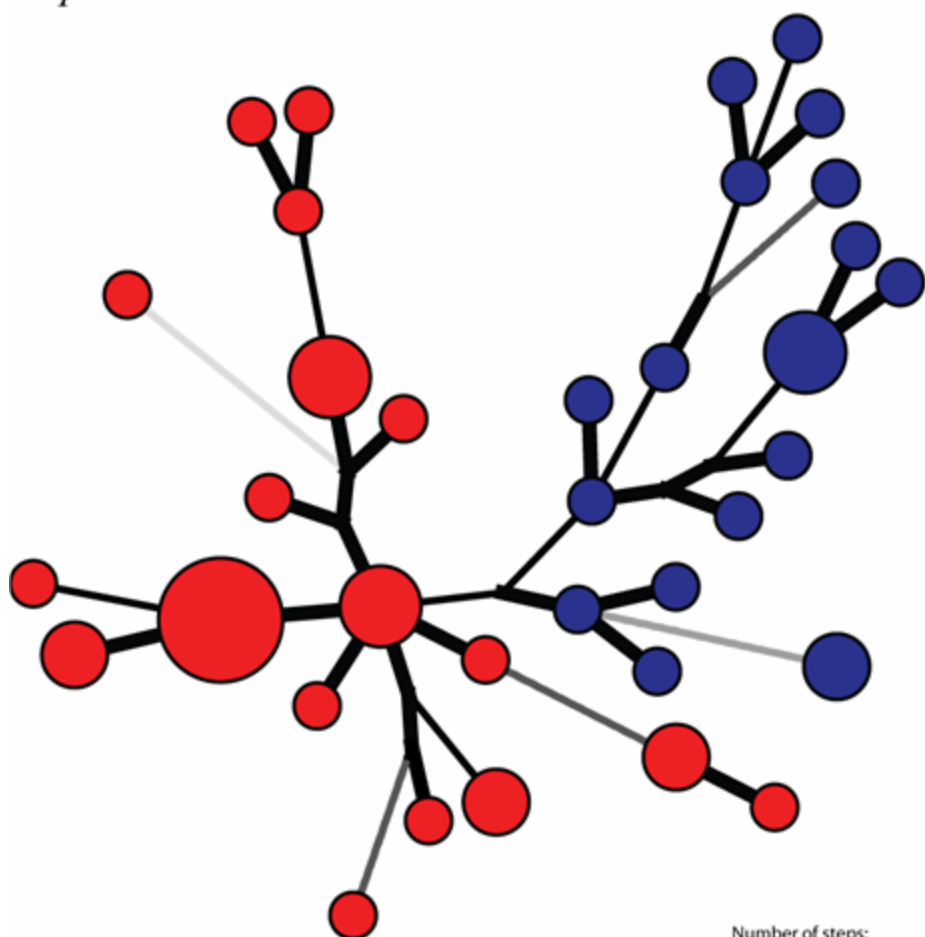

Number of steps:

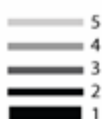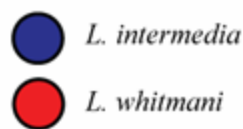

*zetacop*

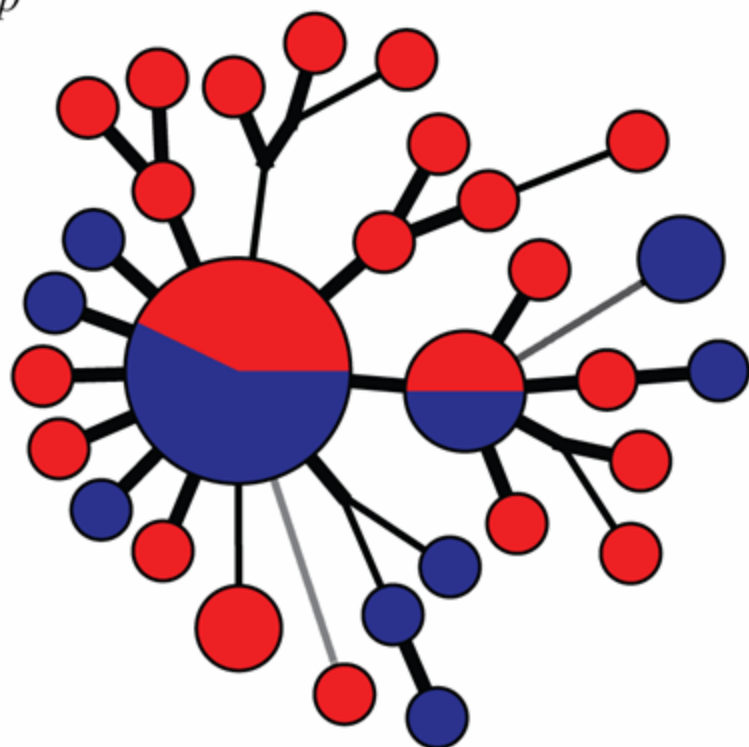

Supplement: Additional file 3 — Haplotype networks of RpL17A and zetacop whole fragments. The figure shows minimum spanning trees of RpL17A and zetacop sequences (whole fragments) of L. intermedia (blue) and L. whitmani (red). The circles are proportional to the haplotype frequencies and the black and grey lines connecting the haplotypes represent the number of mutational steps. [file 1471-2148-8-141-S3.pdf]
